# Major bacterial lineages are essentially devoid of CRISPR-Cas viral defence systems

**DOI:** 10.1038/ncomms10613

**Published:** 2016-02-03

**Authors:** David Burstein, Christine L. Sun, Christopher T. Brown, Itai Sharon, Karthik Anantharaman, Alexander J. Probst, Brian C. Thomas, Jillian F. Banfield

**Affiliations:** 1 Department of Earth and Planetary Science, University of California, Berkeley, Berkeley, California 94720, USA; 2 Department of Microbiology and Immunology, Stanford University School of Medicine, Stanford, California 94305, USA; 3 Department of Plant and Microbial Biology, University of California, Berkeley, Berkeley, California 94720, USA; 4 Department of Environmental Science, Policy and Management, University of California, Berkeley, California 94720, USA

## Abstract

Current understanding of microorganism–virus interactions, which shape the evolution and functioning of Earth's ecosystems, is based primarily on cultivated organisms. Here we investigate thousands of viral and microbial genomes recovered using a cultivation-independent approach to study the frequency, variety and taxonomic distribution of viral defence mechanisms. CRISPR-Cas systems that confer microorganisms with immunity to viruses are present in only 10% of 1,724 sampled microorganisms, compared with previous reports of 40% occurrence in bacteria and 81% in archaea. We attribute this large difference to the lack of CRISPR-Cas systems across major bacterial lineages that have no cultivated representatives. We correlate absence of CRISPR-Cas with lack of nucleotide biosynthesis capacity and a symbiotic lifestyle. Restriction systems are well represented in these lineages and might provide both non-specific viral defence and access to nucleotides.

## Abstract (web summary)

It is thought that CRISPR-Cas systems, which confer acquired immunity to phage and archaeal viruses, are widespread among bacteria and archaea. Here, Burstein *et al.* show that entire lineages of uncultivated microorganisms are essentially devoid of CRISPR-Cas systems.

## Graphical abstract

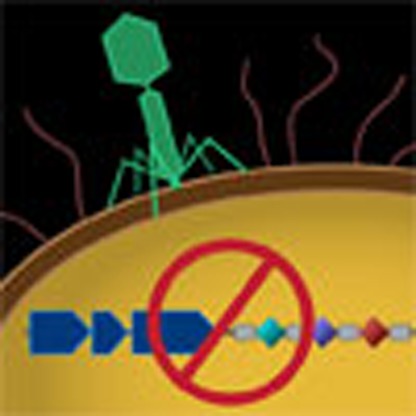

Microorganisms play fundamental roles in the functioning of the biosphere, yet their existence depends on their ability to resist viral predation. CRISPR (clustered regularly interspaced short palindromic repeats) -Cas (CRISPR associated) systems are common defence mechanisms that confer bacteria and archaea with acquired immunity to viruses. The two main parts of the system are a CRISPR array, composed of spacers matching foreign DNA flanked by repeats, and an operon of *cas* genes that encode for proteins that process the CRISPR array and cleave DNA targeted by the spacers^1^,^2^,^3^. Due to its high specificity and programmable nature, CRISPR-Cas has been harnessed to develop a powerful new genome editing technology^4^,^5^,^6^. Current knowledge of the frequency and distribution of CRISPR-Cas is based primarily on genomes of isolated microorganisms^3^,^7^. Here, we expand the investigation of the prevalence, variety and taxonomic distribution of these systems to include more than 40 major lineages of uncultivated bacteria and archaea. We also investigate CRISPR-Cas locus transfer over large evolutionary distances and the presence of alternative viral resistance mechanisms. Finally, we consider possible implications for a link between symbiotic lifestyles and absence of this system.

## Results

### Sampling of microbial and viral populations in groundwater

We studied groundwater filtrates collected as part of a field experiment designed to reproduce conditions that enriched for microbes from lineages that are referred to as Candidate Phyla (CP) because they lack isolated representatives^8^. Over 14 weeks, acetate was added to groundwater in an aquifer adjacent to the Colorado River, near the town of Rifle, Colorado, USA^9^. Twelve samples were collected before, during and after acetate addition. DNA was extracted from cells recovered on serial 0.2 μm (post 1.2 μm) and 0.1 μm (post 0.2 μm) filters and sequenced using Illumina HiSeq technology. In total, we generated more than 250 Gbp of DNA sequence information. The 0.1 μm filtrate samples were of particular interest, as these fractions were enriched in CP bacteria, uncultivated archaea and viruses^9^,^10^,^11^. The acetate manipulation shifted community composition over time, providing access to a wide variety of microorganisms and viruses. We reconstructed 867 circularized viral genomes (614 non-redundant sequences) and more than 57 Mbp of linear or partial virus sequences. Further, we sampled 1,724 microbial genomes that, according to analyses of rRNA genes and phylogenetically informative proteins, originated from members of over 40 phyla, including at least 20 CP^9^,^11^ (Supplementary Data 1). Essentially all of the sampled organisms are from previously unstudied lineages, novel at the genus or higher taxonomic levels.

### CRISPR-Cas systems are less prevalent than expected

CRISPR arrays were identified within the reconstructed genomes (Supplementary Data 2), and their spacers were used to search the metagenomic data set for their viral targets. Given the coexistence of viruses and their potential microbial hosts in the sampled groundwater ecosystem, we anticipated that a large fraction of the CRISPR spacers would match viral sequences. Within the microbial draft genomes we identified CRISPR arrays with 156 distinct repeats harbouring 3,874 unique spacer sequences. Despite the large sampling of viral sequences, only 172 distinct CRISPR spacers (<5%) could be assigned to targets with high probability (≥90% identity). Further, only 14 circularized viral genomes (<3%) were targeted by a spacer. These findings suggested that CRISPR-Cas systems might be less important in environmental microbial communities than expected based on current understanding of the prevalence of this defence system.

**Figure 1:**
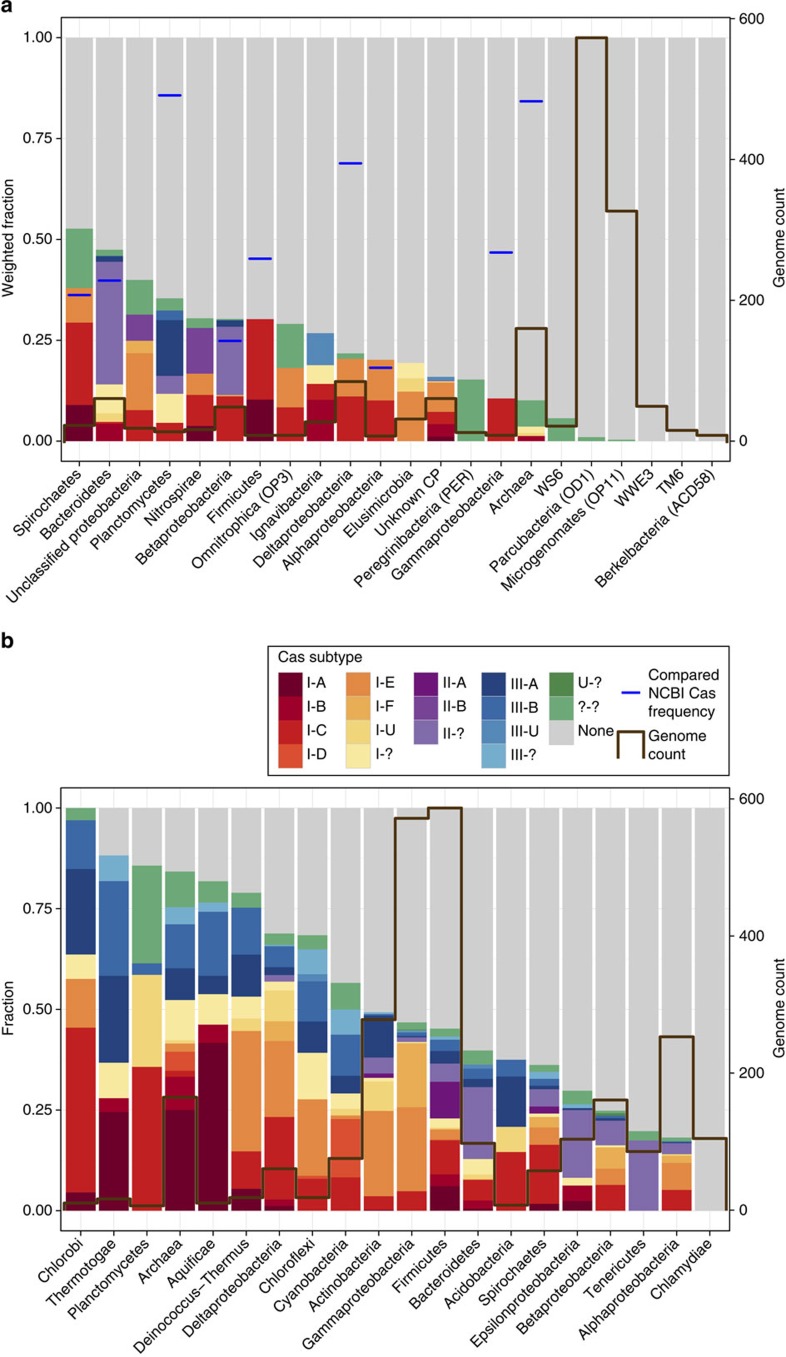
The frequency of *cas* operons in different lineages. (**a**) Rifle groundwater metagenomic data sets and (**b**) NCBI complete bacterial and archaeal genomes. Only lineages with more than ten genomes are presented. Column heights are normalized for genome completeness, column colours represent CRISPR-Cas type, and the bold brown line represents the number of genomes. Blue vertical lines in **a** represent the fraction of genomes with CRISPR-Cas among NCBI complete genomes for applicable lineages. Two superphyla and four phyla in the groundwater samples, including the two most abundant ones, superphyla Parcubacteria (OD1), and Microgenomates (OP11), have an extremely low incidence of *cas* operons.

We next identified *cas* operons using hidden Markov models built based on previously described profiles for the encoded Cas proteins^7^. The *cas* operons were present in merely 166 out of 1,724 draft genomes: in 9.7% of the sampled bacteria and in 9.3% of the archaea (Fig. 1a and Supplementary Data 3). In comparison, previous analyses based primarily on sequences from cultivated microorganisms indicate CRISPR-Cas occurrence in 40% of bacteria and in 81% of archaea^3^,^7^. The difference between the previously reported incidence rate and that found here could arise to a certain extent from the partial nature of the draft genomes recovered from the metagenomic samples. To account for this, we normalized for genome incompleteness, as reflected by the inventory of expected single-copy genes^9^,^12^. After this correction, the frequency at which CRISPR-Cas systems were detected in microorganisms from the studied subsurface system was still substantially below current expectation: 10.4% in bacteria and 10.1% in archaea. We calculated the probability that the difference between the observed occurrence rate of bacterial *cas* operons and the expected occurrence rate based on previous reports is due to genome incompleteness. The probability that *cas1* was present in 40% of bacterial genomes but missed in 29.6% of the cases due to genome incompleteness was estimated to be <10^−90^ (using a conservative estimation; see Methods). These results are compelling because they are based on more than 1,700 draft genomes, an unprecedentedly large environmental data set.

### Entire bacterial phyla essentially lack CRISPR-Cas

Strikingly, CRISPR-Cas systems were absent in almost all complete and draft genomes of organisms from four bacterial phyla and two superphyla, most of which are part of the Candidate Phyla Radiation (CPR)^11^. Specifically, they were found in only 1.4% of over 354 high quality draft genomes (≥80% complete) from organisms assigned to the superphyla Parcubacteria (OD1) and Microgenomates (OP11), and the phyla WWE3, Berkelbacteria (ACD58), WS6 and TM6 (which is not part of the CPR). We do not attribute the absence of these proteins to sequence divergence since our model-based approach was able to identify *cas* operons in those lineages in a few cases. Further, we considered CRISPR-Cas to be present even if only a CRISPR array was found. To rule out the possibility that incorrect gene predictions obscured identification of *cas* operons, we confirmed this analysis using six-frame translations of all genome sequences. This did not reveal any additional operons. The low incidence of CRISPR-Cas systems was not typical for other bacterial groups sampled in this study (Fig. 1a). In fact, we found CRISPR-Cas systems in 31.9% of the sampled bacterial genomes belonging to the phyla that were represented in the last major CRISPR-Cas survey^7^. This is close to the 40% occurrence rate reported in that survey, indicating that the low abundance of CRISPR-Cas we observe is primarily due to the consideration of novel genomes from multiple phyla that were not included in previous surveys.

Samples from additional environments were examined to determine whether the same lineages lack CRISPR-Cas systems in those environments as well. We investigated genomes (≥70% complete) reconstructed from metagenomic samples taken at Crystal Geyser, a deep subsurface CO_2_-saturated cold-water geyser located on the east bank of Green River, UT, USA. Out of 42 genomes of Parcubacteria, Microgenomates, WWE3 and Berkelbacteria species recovered from this site, only three (7.1%) had CRISPR-Cas (Supplementary Data 4). We further analysed all 32 publically available draft and high quality genomes from organisms belonging to these lineages. These organisms were sampled from a variety of environments, including a hospital sink biofilm, a deep-sea hydrothermal vent, a geothermal spring, a deep underground mine and more (Supplementary Data 4). None of these genomes encoded for a CRISPR-Cas systems. Collectively, these results support our finding that major CP lineages are essentially devoid of CRISPR-Cas defence systems.

To place these results in context, we used the same methods to perform an updated survey that included all 2,740 publically available complete bacterial and archaeal genomes from NCBI (Supplementary Data 5) to determine the incidence of CRISPR-Cas systems. The analysis indicated an incidence rate of 40.6% for bacterial genomes and 79.4% for archaeal genomes (Supplementary Data 6), in agreement with previous reports^3^,^7^. Interestingly, we did not detect any *cas* operons or CRISPR arrays in the Chlamydiae phylum. It has been previously reported that Chlamydiae have no type II CRISPR-Cas systems^13^, but here we report that all 105 complete genomes from organisms representing this phylum lack any type of CRISPR-Cas system. This finding reinforces the conclusion that these defence systems can be essentially absent from entire bacterial lineages. Overall, our analyses suggest that the distribution of the CRISPR-Cas immune system is highly uneven across the different branches of the bacterial tree.

### Evidence for horizontal transfer of CRISPR-Cas across phyla

**Figure 2:**
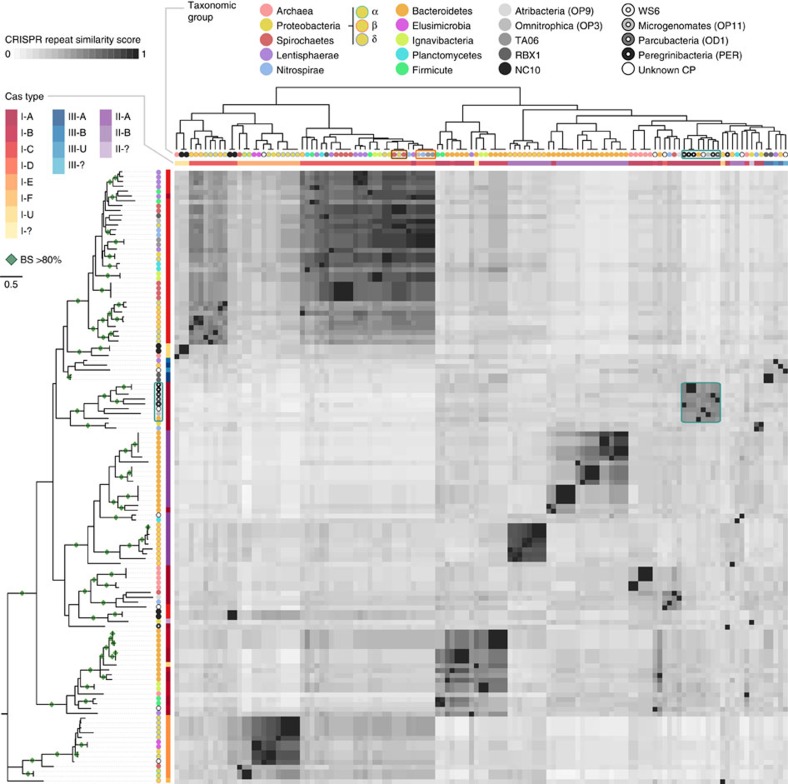
Cas1 phylogeny versus repeat similarity in CRISPR-Cas retrieved from Rifle groundwater samples. Cas1 phylogeny (left) compared with the similarity of repeats in associated CRISPR arrays (top). Coloured circles on the branch tips represent taxonomic affiliation. Colour strip represent CRISPR-Cas type. Heat map shades correspond to full-length repeat similarity. Genomes from different phyla with identical repeats are marked by brown and orange boxes. The cyan box highlights a cluster of genomes with closely related Cas1 proteins and similar repeats, which includes one Bacteroidetes genome and most of the genomes from CPR organisms that have a CRISPR-Cas system. Green diamonds mark branching with bootstrap values (BS) >80%.

The near-absence of CRISPR-Case across entire phyla is intriguing given the evidence that these systems are readily transferred between microbes, potentially even across phylum boundaries^14^,^15^,^16^,^17^,^18^. In support of cross-phyla transfer, we find that the phylogeny of Cas1 proteins is inconsistent at the phylum level with the taxonomy of the organisms that contain the system (Fig. 2). The results for Cas1 and repeat sequences are congruent, as expected if simultaneous transfer of both locus components is required to maintain CRISPR-Cas function. Analyses of both Cas1 and the repeats suggest that CRISPR-Cas was transferred to CPR organisms, but in most cases selected against, as indicated by partial *cas* operons (Supplementary Data 3) and the restricted distribution of the system among these organisms. Notably, we identified 100% identical repeat sequences in organisms from three different bacterial phyla (Fig. 2). Overall, these findings demonstrate that horizontal transfer of the CRISPR-Cas indeed occurs frequently and even among organisms from different phyla, but in certain phyla it is selected against.

CRISPR-Cas systems are classified into different types based on the inventory of Cas proteins^7^. As in genomes from cultivated organisms, the least common system type in Rifle groundwater samples was type II. This type is characterized by minimal *cas* operons and the presence of Cas9, a protein of great biotechnological interest. Cas9 was found in organisms from only four bacterial phyla, and in 13.6% of all *cas* operons. Due to the interest in Cas9 we provide sequences and phylogeny of Cas9 proteins identified in Rifle metagenomic samples (Supplementary Data 7; Supplementary Fig. 1).

### Lack of CRISPR-Cas in symbionts

We considered the possibility that CRISPR-Cas systems are absent from specific phyla because these organisms experience little viral predation. However, we identified the presence of integrated viral genes, as well as alternative viral defence systems, mostly restriction–modification^19^ and abortive infection^20^, in the CP lineages lacking CRISPR-Cas (Supplementary Fig. 2A). Further indicating ongoing viral predation, a recent cryo-TEM characterization of 0.1 μm filtrates from the current study detected virus particles directly associated with the surfaces of the ultra-small cells, primarily from Parcubacteria (OD1), Microgenomates (OP11) and WWE3 (ref. 10). In addition, several Chlamydia viruses have been reported^21^. Given evidence that these organisms experience viral predation, we conclude that CRISPR-Cas is probably selected against because the costs of maintaining the systems^22^,^23^ outweighs the benefit of the defence it provides.

One of the potentially detrimental costs associated with CRISPR-Cas viral defence is autoimmunity, which arises when CRISPR arrays acquire spacers that target the host genome^24^. Interestingly, two of the nine arrays found in CP with low CRISPR-Cas incidence had spacers with significant identity to the host bacterial genome. In one case, we found a spacer 100% identical to a segment of the 16S ribosomal RNA gene. This observation indicates that avoidance of self-targeting might be ineffective in these organisms, thus rendering CRISPR-Cas toxic to these cells.

**Figure 3:**
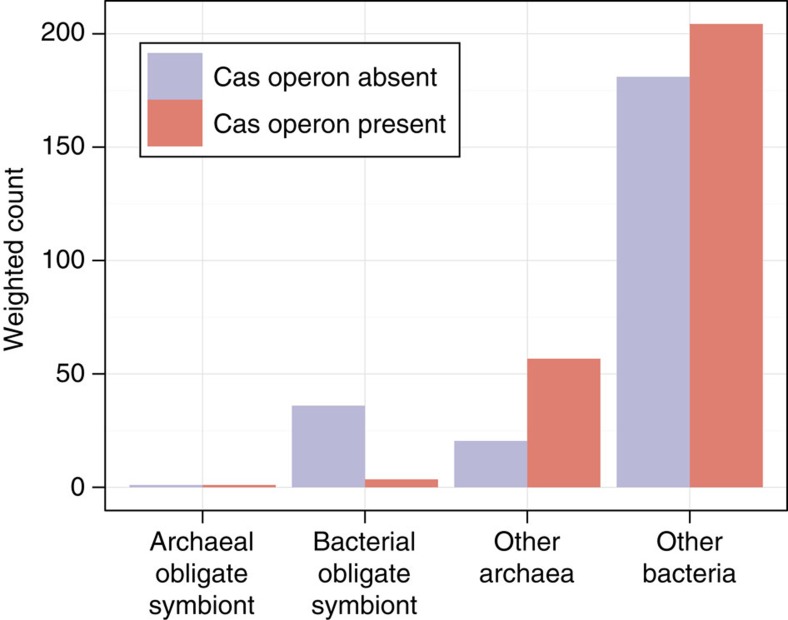
Association between obligate symbiotic lifestyles and presence of CRISPR-Cas among well-studied bacteria and archaea. Existence of CRISPR-Cas (purple: absent; red: present) among 1,703 sequenced organisms, including 203 obligate symbionts (two archaea; 201 bacteria) and 1,500 organisms that are not obligate symbionts (164 archaea and 1,336 bacteria). To address sample biases, each genome was assigned a weight inversely proportional to the number of genomes represented in the genus. Thus, each genus received a total count of 1 on the *y* axis. Even when considering the differences in CRISPR-Cas abundance between bacteria and archaea, a significant association is found between obligate symbiotic lifestyles and presence of CRISPR-Cas (*P* value <1.6 × 10^−8^, ANOVA).

Alternatively, it is possible that CRISPR-Cas systems are not retained due to shared features of the organisms that lack them. Notably, organisms from CPR, TM6 and Chlamydiae have very small genomes (∼1 Mbp) and they are inferred to be obligate symbionts^11^,^25^,^26^,^27^,^28^,^29^. Thus, we hypothesized that selection against CRISPR-based viral defence is a general feature of symbionts. To test this, we compared the presence of CRISPR-Cas among well-studied free-living and obligate symbionts (genus-level analysis, see Methods and Supplementary Data 8). We found a significant association (*P* value<1.6 × 10^−8^ ANOVA) between absence of CRISPR-Cas and obligate symbiotic lifestyles (Fig. 3). To support previous reports of symbiotic lifestyle of CPR^11^,^25^,^26^,^27^,^28^ organisms, we surveyed 528 high-quality genomes from the groundwater samples (estimated completeness ≥80%) for presence of fatty-acid and nucleotide biosynthesis. All the organisms from phyla lacking CRISPR-Cas had no fatty-acid initiation or elongation pathways (Supplementary Fig. 3), suggesting they acquire fatty acids from external sources. An analysis of the co-occurrence of nucleotide biosynthesis pathways with viral defence systems in a combined set of quality genomes from NCBI and the groundwater samples, revealed that CRISPR-Cas systems are extremely underrepresented in organisms without nucleotide biosynthesis pathways (*P* value <10^−116^, Fisher exact test, Supplementary Fig. 4A). Curiously, among organisms lacking nucleotide biosynthesis pathways, restriction systems were more represented in CPR organisms (78%) compared with non-CPR organisms (62%). Similarly, the number of restriction enzymes encoded in the genomes of CPR organisms lacking nucleotide biosynthesis is significantly higher than in non-CPR organisms lacking these pathways (*P* value<10^−39^, Mann–Whitney test, Supplementary Fig. 4B).

## Discussion

Entire lineages of uncultivated organisms, which have been shown to be widespread across many environments^10^,^30^, are essentially devoid of CRISPR-Cas. We suggest three possible explanations for this observation. The first explanation arises from their likely obligate symbiotic lifestyle, an inference based on the lack of biosynthesis pathways for basic cellular building blocks, and supported by a recent experimental study showing that a CPR organism from the Saccharibacteria (TM7) phylum is an obligate epibiont of an Actinobacterium^31^. A lifestyle that requires close association of multiple symbiont cells with a host cell could lead to higher phage densities, which have been experimentally shown by Westra *et al.*^22^ to cause selection against CRISPR-Cas. Second, the efficacy of CRISPR-Cas depends on the viral mutation rate and spacer incorporation frequency^32^. Given their low ribosome contents^10^, CPR probably have extremely slow growth rates, and may not be able to mount a response to an infection event fast enough or to acquire spacers quickly enough for the CRISPR-Cas system to be effective. Finally, the CRISPR-Cas system may be selected against due to autoimmunity^24^,^33^: self-targeting spacers have been incorporated, possibly due to lack of mechanisms to distinguish self from other^34^ in these small genomes.

Genes for restriction enzymes, which are overrepresented in these genomes compared with symbionts from other lineages, may provide access to nucleotides by degrading exogenous DNA, including that derived from inactivated viruses. Lipids may be acquired from the hosts of these organisms, as was previously suggested for the intracellular pathogen *Renibacterium salmonarum*^35^. Combined, these findings raise the intriguing possibility of symbioses due to mutual benefits that arise from membrane sharing and DNA acquisition from degraded viruses. Specifically, CPR that are extracellular symbionts of bacteria, and derive cell wall material from their host bacteria may retain the surface-associated viral recognition targets of the host cell. It is possible then that CPR bacteria provide a service to their hosts by serving as ‘viral decoys', lowering the host viral load, while taking advantage of the degraded viral DNA as a nucleotide source.

## Methods

### Sample collection, sequencing, assembly and binning

Groundwater was pumped from an aquifer at the Rifle Integrated Field Research Challenge (IFRC) site, near the town of Rifle, CO, USA at six time points (A–F) between August and December 2011. Samples were taken before, during and after acetate addition to the aquifer as part of a biostimulation experiment aimed at enriching organisms from little studied CP^9^,^10^,^11^. Groundwater was serially filtered by 1.2, 0.2 and 0.1 μm filters. The 0.1 and 0.2 μm filters were flash-frozen in a dry ice/ethanol bath on site. DNA was extracted from the filters using the PowerSoil DNA Isolation Kit (MO-BIO), with two modifications to the protocol: DNA elution was done in 50 μl Tris buffer; and DNA concentration was done by adding 500 μl of 3 M sodium acetate (pH 5.2, adjusted with glacial acetic acid) and 13.75 μl of glycogen (20 g l^−1^) to 5 ml of DNA elution, followed by 10 ml of 100% sterile filtered ethanol.

Extracted DNA was sequenced by the Joint Genome Institute on the Illumina HiSeq 2000 platform producing 150 bp paired-end reads with a targeted insert size of 500 bp. All the samples combined produced more than 200 Gbp represented by ∼1.6 × 10^9^ reads. Reads were trimmed using Sickle^36^, and then assembled into scaffolds with IDBA_UD^37^. Scaffold coverage was calculated by mapping reads to the assembly using Bowtie2 (ref. 38). Gene prediction was performed by Prodigal (−p meta)^39^ for all scaffolds longer than 5 kbp. Gene annotations were determined based on reciprocal best usearch (−ublast)^40^ hits against UniRef90 (ref. 41) and KEGG^42^ databases. One-direction best hit was used when no reciprocal hits were found. Predicted genes were also scanned for known domains and other protein signatures using InterProScan^43^.

Initial clustering of scaffolds into genomic bins was performed using the ABAWACA binning algorithm (https://github.com/CK7/abawaca/tree/master), which used scaffold taxonomic affiliations, time-series abundance patterns, and nucleotide frequencies as measurements to inform binning. Manual refinement of draft genome bins followed. Each bin was evaluated based on coverage, GC content and phylogenetic profile of gene best hits. Genome completeness was evaluated based on inventories of 51 single-copy genes for bacterial genomes^12^, and 54 single-copy genes for archaeal genomes^9^. We recovered a total of 1,724 partial and complete genomes that could be associated to a microbial lineage. The genomes are detailed in Supplementary Data 1.

Crystal Geyser data was assembled as described above from reads of two previously published metagenomic samples acquired from a CO_2_-driven geyser, located 6 km south of the town of Green River, Utah, USA, on 8 November 2009 (BioSamples SAMN02419149 and SAMN02419150)^44^. Time-series abundance was determined using additional samples acquired from the site 32 h earlier (6 November 2009; BioProject PRJNA297582). The scaffolds of the two samples were individually binned using ABAWACA, time-series ESOMs (scaffolds>3 kb)^12^, and tetranucleotide-frequency based ESOMs (scaffolds >5 kb, 10 kb window)^45^.

### Publically available complete bacterial genomes

Supplementary Data 5 contains the accessions and NCBI Taxonomy IDs for all complete bacterial genomes that were acquired from NCBI's bacterial genomes ftp site (ftp://ftp.ncbi.nih.gov/genomes/Bacteria/).

### Viral sequences

Circular viral genomes were identified by searching for scaffolds that could be circularized using paired reads mapped to either end of the scaffold. For each data set we first used Bowtie2 (ref. 38) with parameters ‘-q -n 2 -e 200 --best' to map the raw reads to all assembled scaffolds longer than 5 kbp. Next, we searched for scaffolds whose ends were connected by at least five paired-end reads, and for which no connection to any other scaffold could be identified. Genomes were classified as viral based on existence of viral genes (specified below) and lack of plasmid-specific genes. Genomes that were≥99% identical over≥99% of the sequence were considered to belong to the same virus population.

Additional scaffolds were considered viral if they satisfied the following: (a) could not be assigned to any bacterial lineages based on protein similarity; (b) contained two or more viral genes, specifically, ORFs annotated with virus-specific terms (‘capsid', ‘phage', ‘terminase', ‘baseplate', ‘base plate', ‘prohead', ‘virion', ‘holing', ‘virus', ‘viral', ‘tape measure', ‘tapemeasure', ‘neck', ‘tail', ‘p22', ‘head', ‘T4'); (c) did not include cellular-specific genes (tRNA synthetases, ribosomal protein, preprotein translocases or DNA gyrase subunit A).

Information regarding integrated viral sequence among NCBI completely sequences bacterial genomes was acquired from PHAST^46^ database at http://phast.wishartlab.com/Download.html.

### CRISPR-Cas detection

All the microbial draft genomes assembled from Rifle groundwater were scanned for CRISPR arrays using CRISPRfinder^47^, and the resulting arrays were manually curated to filter out false positives. Similarity among retrieved CRISPR repeats was calculated based on all versus all full-length comparison of repeats using ‘needle' from the EMBOSS suite^48^. For the heat map presented in Fig. 2, bit scores were normalized based on self-scores. Supplementary Data 3 provides information regarding all the CRISPR arrays identified within the Rifle groundwater genomes.

To identify *cas* operons, hidden Markov models (HMMs) were constructed for each Cas protein using HMMER 3.1b1 (ref. 49), based on multiple sequence alignments from the NCBI CRISPR/Cas website^7^. These Cas HMMs were used to detect significant hits (E-value <10^−10^) among the genomes from both Rifle groundwater and NCBI genomes. The presence of Cas1, or alternatively two adjacent (≤5 ORFs apart) Cas proteins indicated a *cas* operon.

To ensure no *cas* operon was missed due to incorrect gene prediction, six-frame translations of all relevant scaffolds was carried out using ‘transeq' from the EMBOSS suite^48^, and all frames were scanned using the Cas1 HMMs. No additional Cas1 proteins were detected by this search, excluding one partial (<20%) Cas1 protein, which was found on a contig belonging to an uncharacterized candidate phylum.

We estimated a lower bound for the probability that *cas* operons were missed due to genome incompleteness based on the average length of *cas1* as follows. First, the complete genome length was estimated by dividing the number of recovered nucleotides by the estimated genome completeness (see above). We then calculated the probability for randomly missing a segment of *cas1* length (775 nucleotides) given the estimated length of the full genome and the number of nucleotide recovered. This is an underestimation as the length of the CRISPR array was not taken into account and *cas* operons include several additional proteins.

We tested for association between genome lengths and incidence rates of CRISPR-Cas systems and found that, within each phylum, the presence or absence of CRISPR-Cas could not be explained by the genome length (*P* value 0.18, nested ANOVA, Supplementary Fig. 5).

### Phylogenetic analyses

Cas1 phylogeny was based on all Cas1 proteins identified in the Rifle groundwater genomes (scaffolds detailed in Supplementary Fig. 6). Cas9 phylogeny included all the full-length Cas9 proteins available in RefSeq (http://www.ncbi.nlm.nih.gov/refseq/), as well as Cas9 proteins from Rifle metagenomic samples, ORFs and accession numbers are detailed in Supplementary Fig. 7 and Supplementary Data 7. For both Cas1 and Cas9 data sets, amino acid multiple sequence alignments were performed using MAFFT with the ‘linsi' scheme^50^, followed by removal of partial proteins. Maximum-likelihood phylogenies were inferred by RAxML^51^ under the PROTGAMMALG evolutionary model with 100 bootstrap re-samplings.

### Alternative viral defence mechanism

The number of restriction–modification systems^19^ was determined based on the number of identified restriction enzymes (to avoid over-counting, methylation and specificity subunits were not considered). Restriction enzymes were identified based on either similarity (BLAST e-value ≤10^−10^, coverage ≥80%) to restriction enzyme entries of REBASE, the restriction enzyme database^52^, or annotation: considering ORFs including the term ‘restriction' in their annotation, but not one of the following terms (as a complete word): ‘methyltransferase', ‘methylase', ‘methylation', ‘restriction alleviation', ‘antirestriction', ‘M', ‘mod', ‘specificity', ‘S', ‘McrC', ‘hsdS', ‘hsdM', ‘DptF', ‘DptG', ‘dam', ‘ccrM', ‘yhdJ', ‘DNMT1', ‘E2.1.1.113', ‘E2.1.1.72', ‘EC:2.1.1.113', ‘EC:2.1.1.72', ‘ArdA', ‘ArdC' and ‘lar'.

Abortive infection systems^20^ were identified based on Abi-coding genes or two toxin–antitoxin coding genes. ORFs with the term ‘abortive' or ‘abi' were considered Abi-coding genes. Toxin–antitoxin genes were identified based on either BLAST similarity (e-value ≤10^−10^, coverage ≥80%) to entries from the toxin–antitoxin database^53^ or on annotation, including ORFs with one of following terms: ‘antitoxin', ‘anti-toxin', ‘antidote-toxin', ‘addiction module', ‘mazE', ‘mazF', ‘ChpAK', ‘ChpAI', ‘mosT', ‘mosA', ‘yeeV', ‘yeeU', ‘pasB', ‘pasA', ‘pemK', ‘pis', ‘pemI', ‘vapB', ‘vapC', ‘relE', ‘relE4', ‘stbE', ‘prevent-host-death', ‘doc', ‘phd', ‘txe', ‘yoeB', ‘yefM', ‘cogG', ‘metJ', ‘relB', ‘dinJ', ‘yafQ', ‘parE', ‘parDB', ‘parD1', ‘hicA', ‘hicB', ‘hipA', ‘hipB', ‘ccdB', ‘ccdA'; excluding ORFs whose annotation include: ‘topoisomerase', ‘cytochrome', ‘chemotaxis' or ‘motility'.

BREX systems were identified based on the presence of PglZ homologues^54^,^55^, detected either by their annotation (genes annotated as ‘*plgZ*'), or significant hmmsearch^56^ hit (E-value<10^−5^) to Pfam's PglZ HMM (PF08665)^57^.

### Obligate symbiosis analysis

For the 1,703 organisms with a complete genome in NCBI and a record in IMG^58^, we determined whether they are obligate symbionts based on IMG annotation and literature searches (Supplementary Data 7). To detect the significance of the association between the obligate symbiotic lifestyle and the presence of CRISPR-Cas, we used a logistic regression to fit the presence or absence of *cas* operons to the lifestyle of the microorganism. To account for the large sample bias of available genomes we assigned each genome with a weight inversely proportional to the number of genomes sampled from the same genus.

### Nucleotide and fatty acid biosynthesis pathways

Each KEGG^42^ ortholog group was represented by a HMM: the group's proteins were aligned with MAFFT^50^ and HMMs were constructed using hmmbuild from the HMMER suite^56^. The ORFs from each genome were scanned against the ortholog group HMMs, and were assigned an orthologous group according the best HMM hit (with E-value <0.01). The completeness of a given biosynthesis pathway was determined based on the number of stages in the pathway for which genes were detected in the genome in question. Nucleotide biosynthesis was considered to be present in a genome if ≥75% of the combined stages of KEGG modules M00048 (Inosine monophosphate biosynthesis) and M00051 (Uridine monophosphate biosynthesis) were satisfied, and it was considered absent if only a third or less of the stages were satisfied (genomes with more than a third but <75% of the pathways were considered inconclusive and not taken in to account in this analysis). The fraction of the fatty-acid pathway in a genome was determined based on the number of stages whose genes were detected among KEGG modules M00082 (fatty-acid biosynthesis initiation) and M00083 (fatty-acid elongation).

## Additional information

**Accession codes:** Reads and relevant scaffolds from the Rifle Groundwater metagenomes have been deposited in the NCBI BioSample database with accession codes SAMN03202994 to SAMN03203005, and in NCBI BioProject database with accession codes PRJNA298485, PRJNA288027, and PRJNA273161. Crystal Geyser reads and relevant scaffolds are available as part of BioProject PRJNA297582.

**How to cite this article:** Burstein, D. *et al.* Major bacterial lineages are essentially devoid of CRISPR-Cas viral defence systems. *Nat. Commun.* 7:10613 doi: 10.1038/ncomms10613 (2016).

## Supplementary Material

Supplementary Figures Supplementary Figures 1-7

Supplementary Data 1 Genomes reconstructed from Rifle groundwater metagenomic samples.

Supplementary Data 2 CRISPR arrays found in Rifle groundwater genomes - location, size and repeat.

Supplementary Data 3 *cas* operons identified in Rifle groundwater genomes.

Supplementary Data 4 Presence of *cas* operons in organisms from Parcubacteria, Microgenomates, WWE3, Berkelbacteria, WS6 and TM6, sampled from other environments.

Supplementary Data 5 Publically available complete microbial genomes screened for CRISPR-Cas.

Supplementary Data 6 *cas* operons identified in NCBI complete microbial genomes.

Supplementary Data 7 Multiple sequence alignment of Cas9 proteins.

Supplementary Data 8 Obligate symbiosis and presence of *cas* operons in complete microbial genomes.
