# Supplementary material for: Major bacterial lineages are essentially devoid of CRISPR-Cas viral defence systems
Source: Nat Commun. 2016 Feb 3;7:10613. doi: 10.1038/ncomms10613 (PMC4742961; doi:10.1038/ncomms10613)
Supplement: Supplementary Figures — 1-7 [file ncomms10613-s1.pdf]

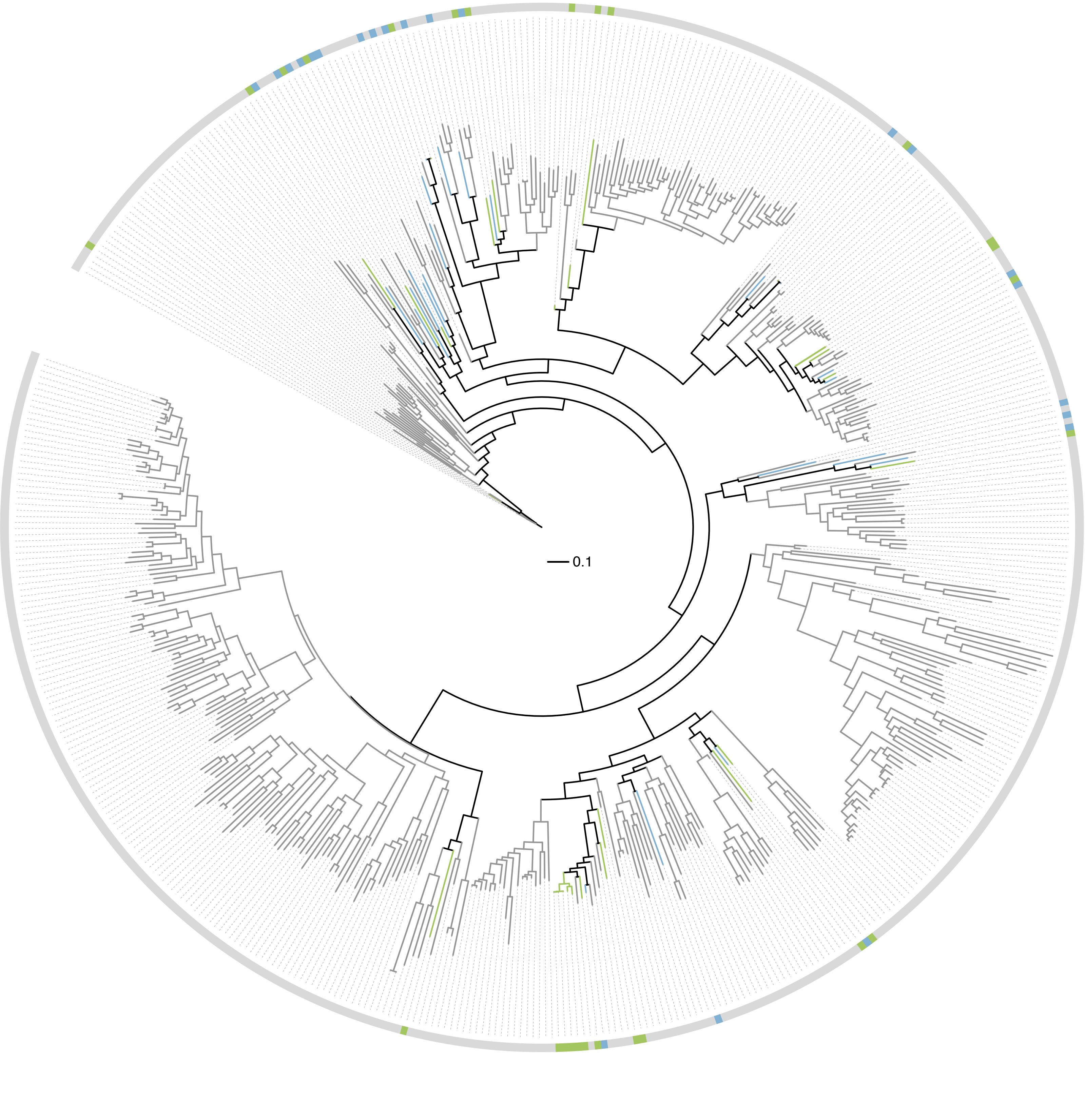

Supplementary Figure 1. Maximum-likelihood Cas9 phylogeny. Cas9 proteins from RefSeq are in grey; ORFs from Rifle groundwater metagenomic data are marked in green; ORFs from other Rifle metagenomic samples are marked in blue. The accession numbers and bootstrap values are detailed in Supplementary Figure 7.

A

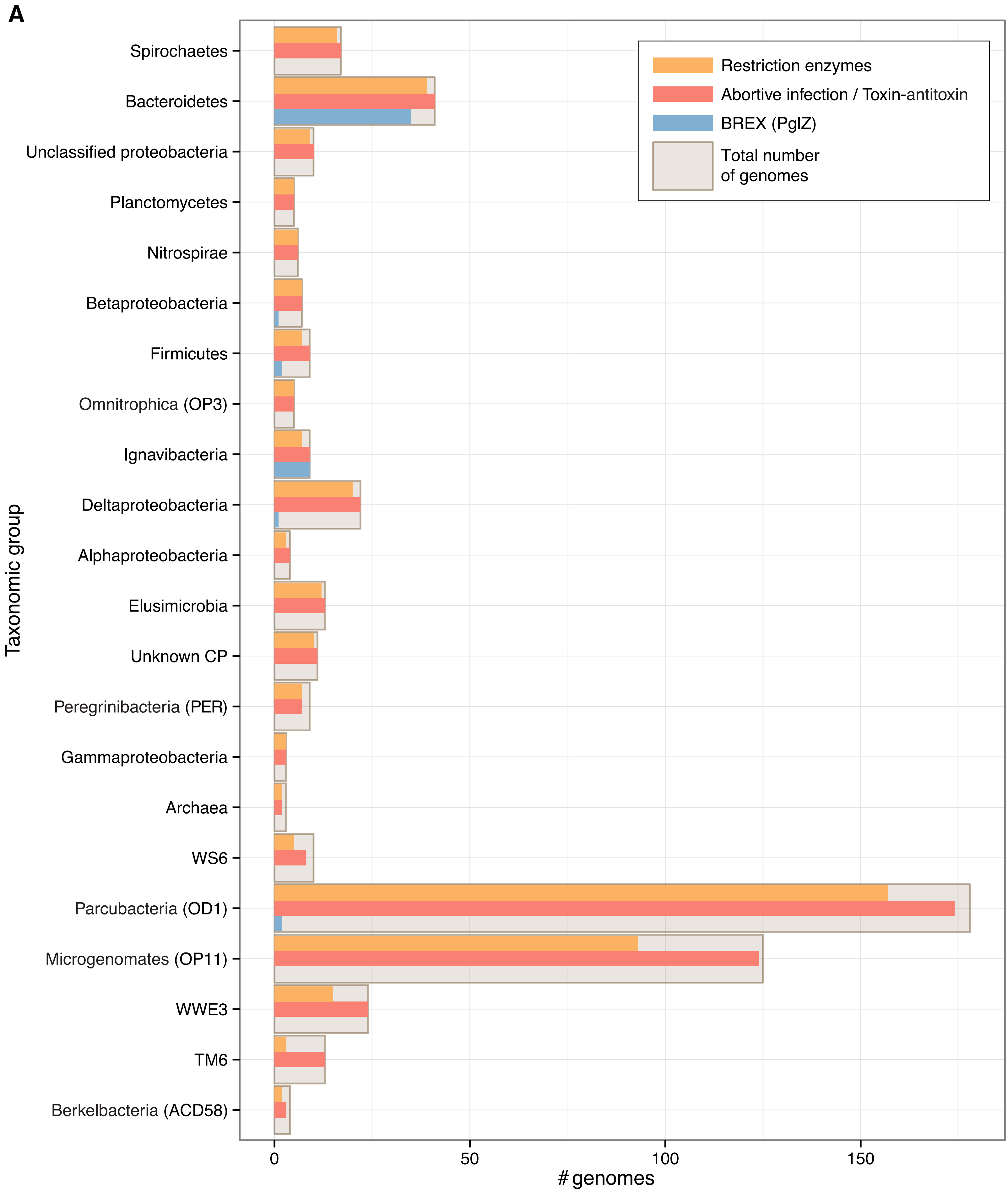

B

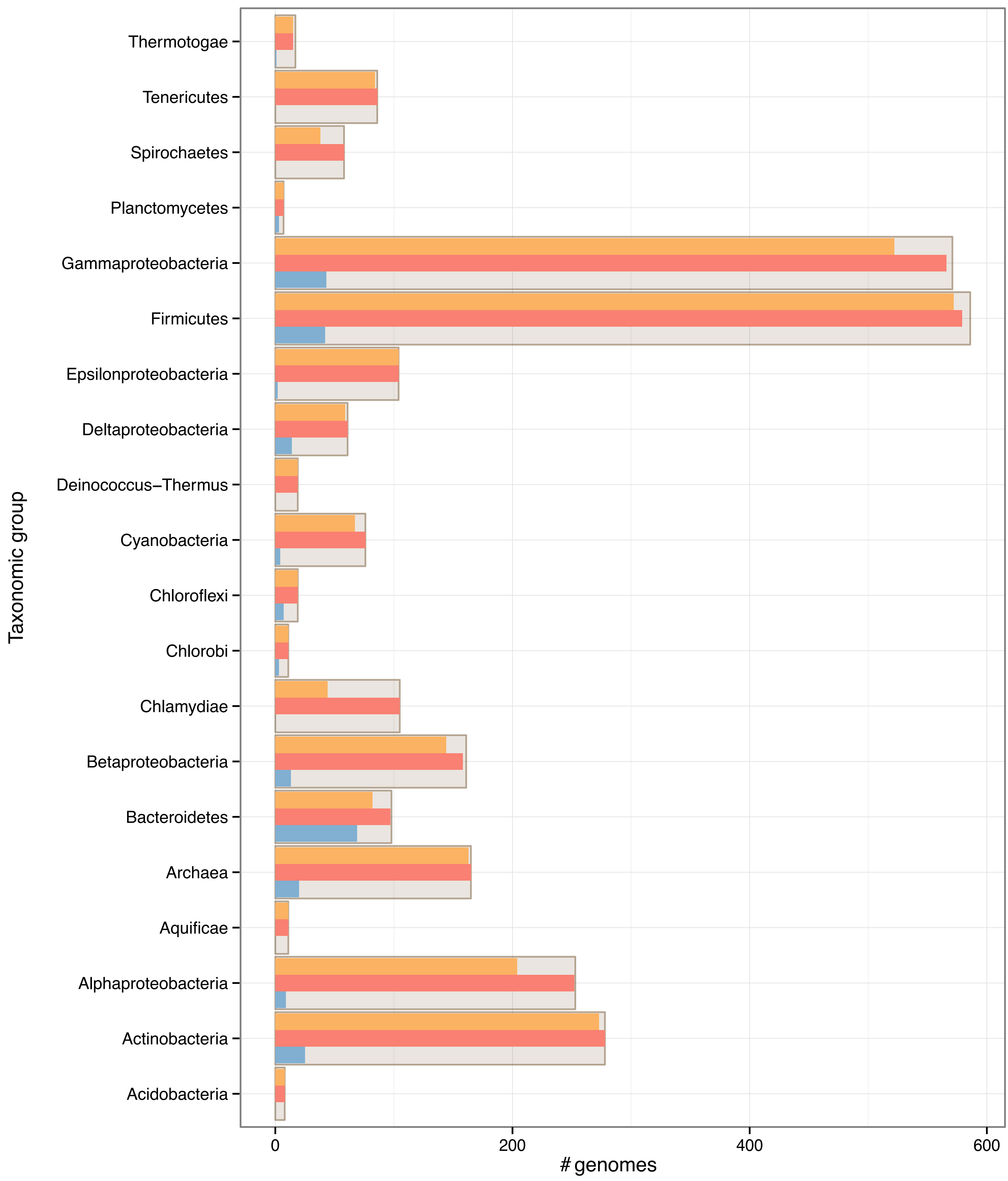

Supplementary Figure 2. Alternative viral defense systems in (A) Rifle groundwater samples, and (B) NCBI complete bacterial genomes. The number of genomes with restriction modification (yellow), abortive infection or toxin-antitoxin (red) and BREX (blue) viral defense system is displayed per taxonomic group. Only genomes with estimated completeness ≥ 80% were considered.

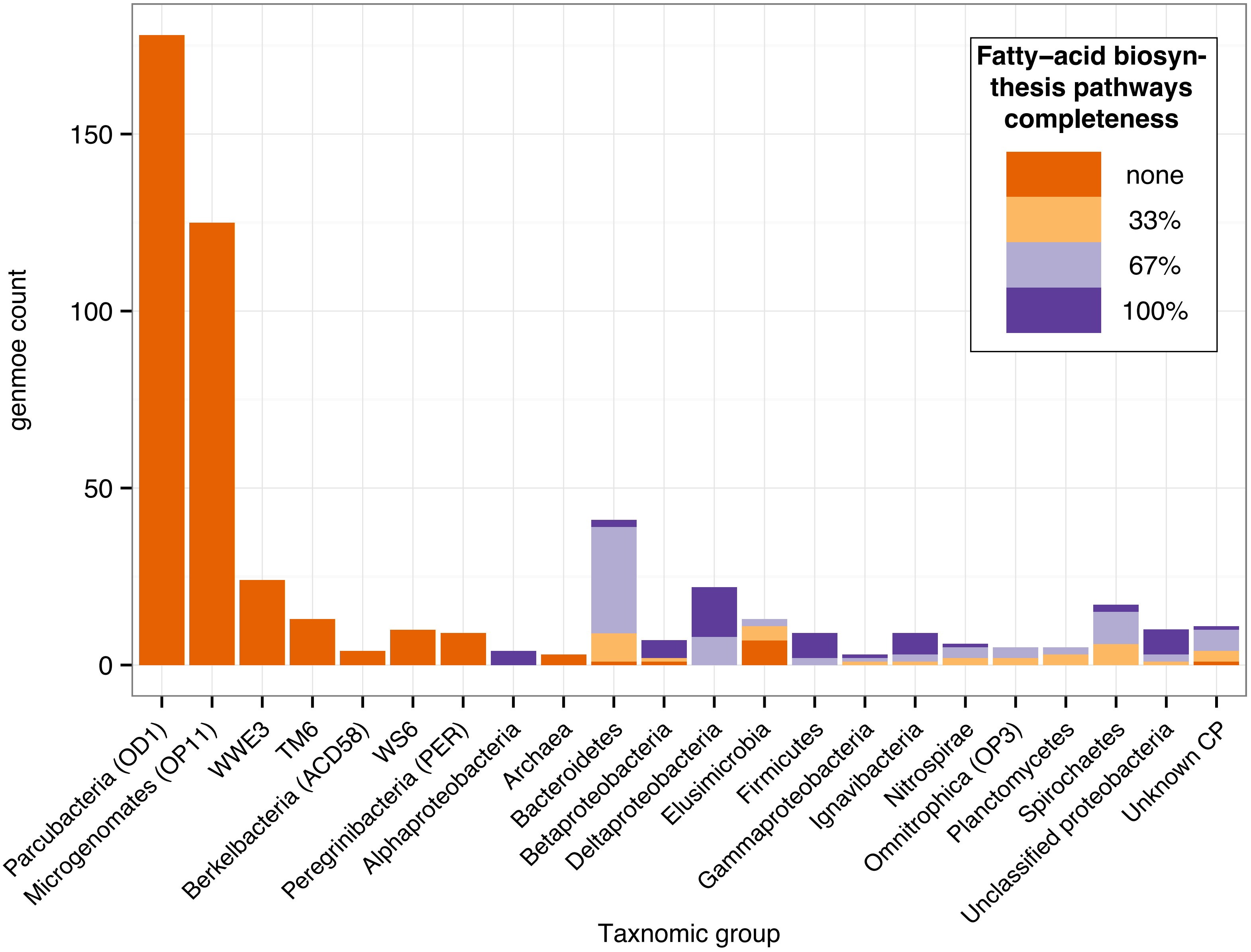

Supplementary Figure 3. Fatty acid biosynthesis pathways among Rifle groundwater genomes. Bar heights represent the number of high quality genomes analyzed in each taxonomic group. Bar colors are according to the completeness of fatty acid initiation and elongation pathways detected. Notably, all bacteria belonging to phyla lacking CRISPR-Cas had no fatty acid biosynthesis pathways.

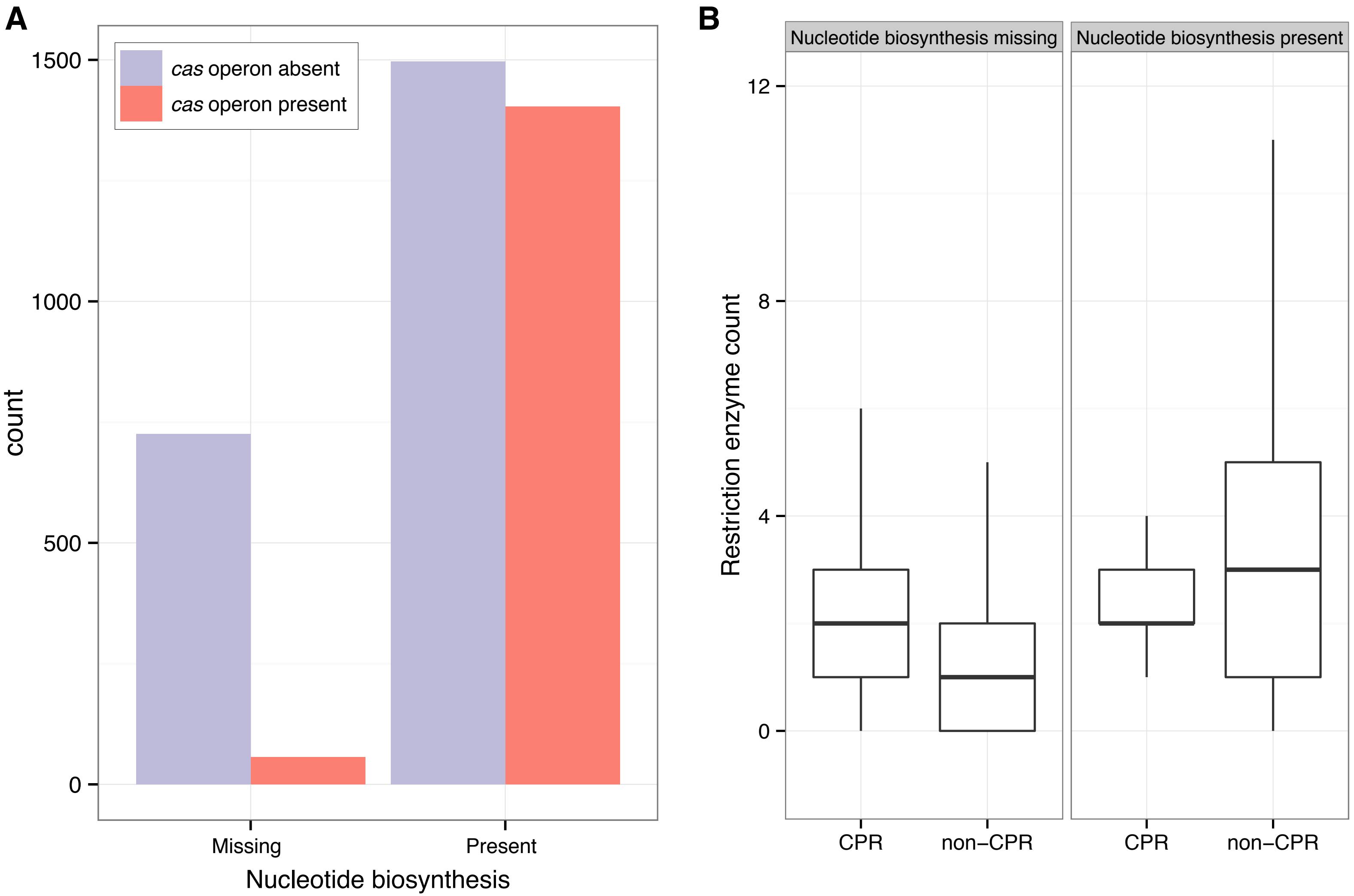

Supplementary Figure 4. Association between nucleotide synthesis pathway and (A) presence of CRISPR-Cas, (B) Restriction systems count. A combined set of all completely sequenced bacterial genomes in NCBI and high quality genomes (estimated completeness ≥ 80%) from Rifle groundwater metagenomic samples were analyzed for association between the presence/absence of nucleotide biosynthesis and two viral defense mechanisms. (A) A significant association was observed between the absence of CRISPR-Cas and absence of nucleotide biosynthesis pathways (p-value < 10^{-116}, Fisher exact test). (B) The number of restriction enzymes encoded by the genomes of CPR organisms lacking nucleotide biosynthesis pathways is significantly higher than the number of restriction enzymes in non-CPR organisms lacking these pathways (p-value 10^{-39} Mann-Whitney test). The box borders represent the 25th and 75th percentiles, and the heavy line within the box is the median number of restriction enzymes.

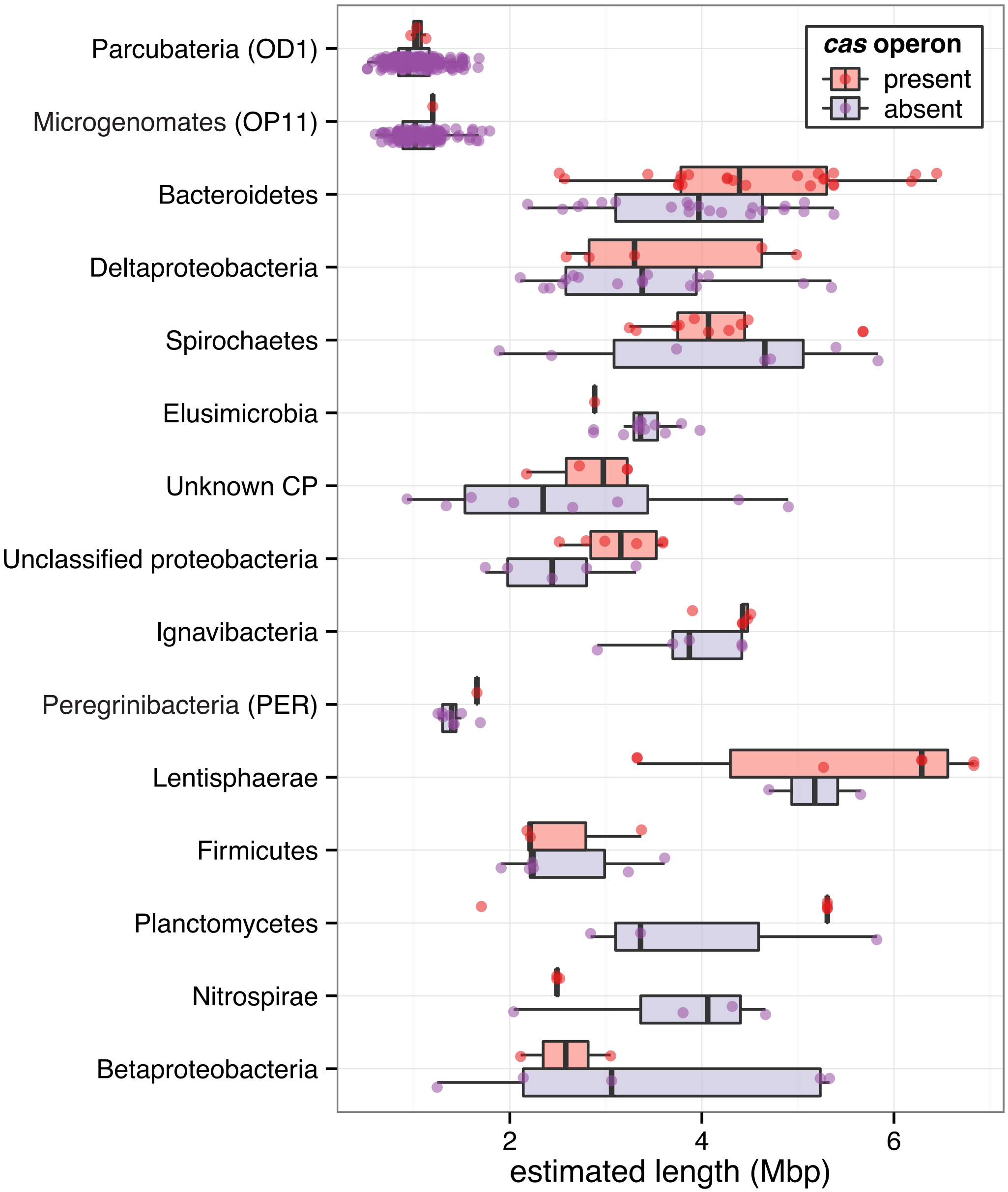

Supplementary Figure 5. Genome length distribution per phylum for genomes assembled from the Rifle groundwater samples. The length of each genome is represented by a red dot if it contains a *cas* operon, or a purple dot if it does not. Box plots with matching colors represent the genomes length distribution. Nested ANOVA indicates that, within each phylum, no significant length differences were found between genomes with CRISPR-Cas system and genomes without it (p-value 0.18).

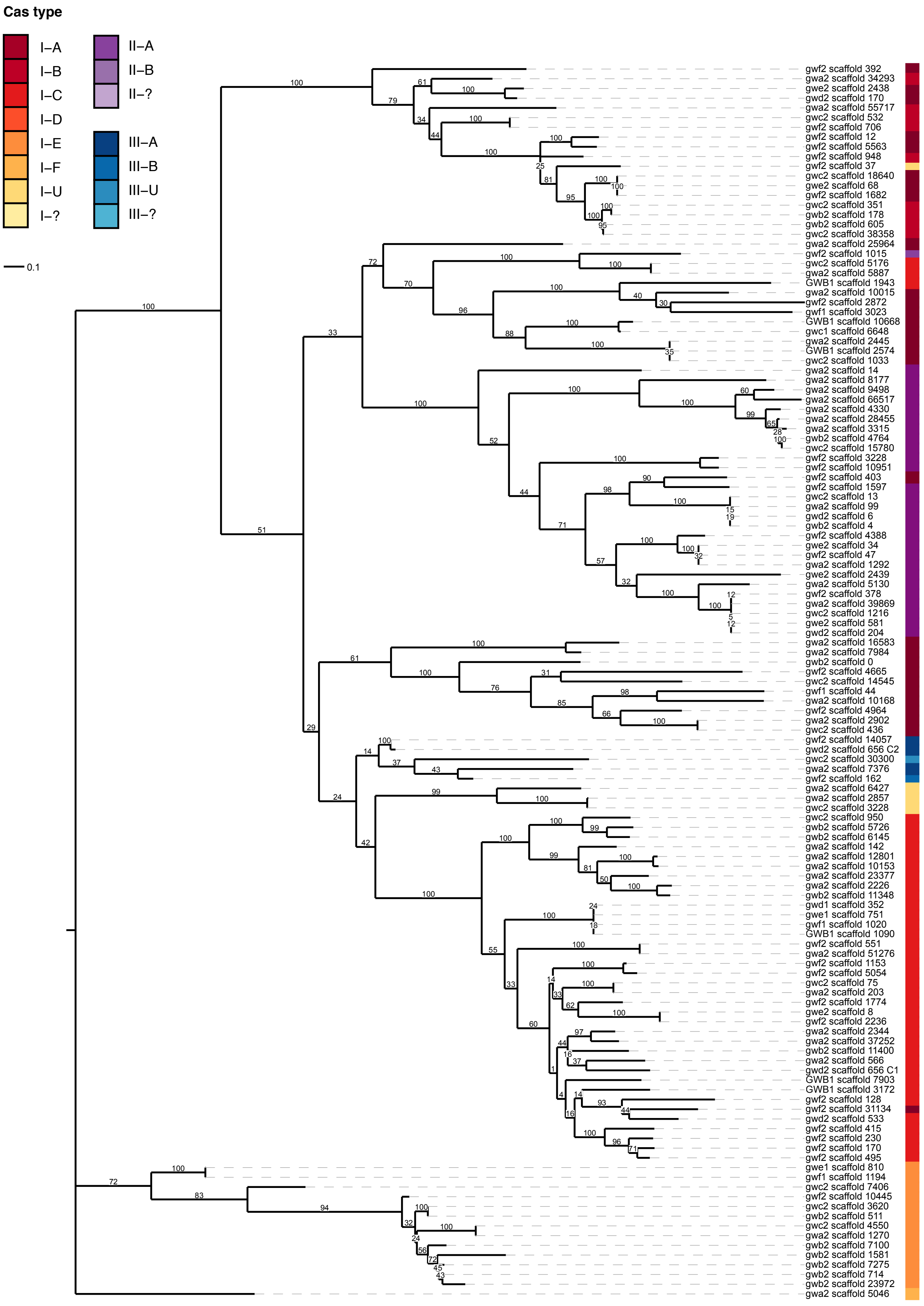

Supplementary Figure 6. Detailed phylogeny of Cas1 proteins from Rifle groundwater. A maximum-likelihood based phylogenetic tree (detailed version of Fig. 2), including scaffolds names and bootstrap values.

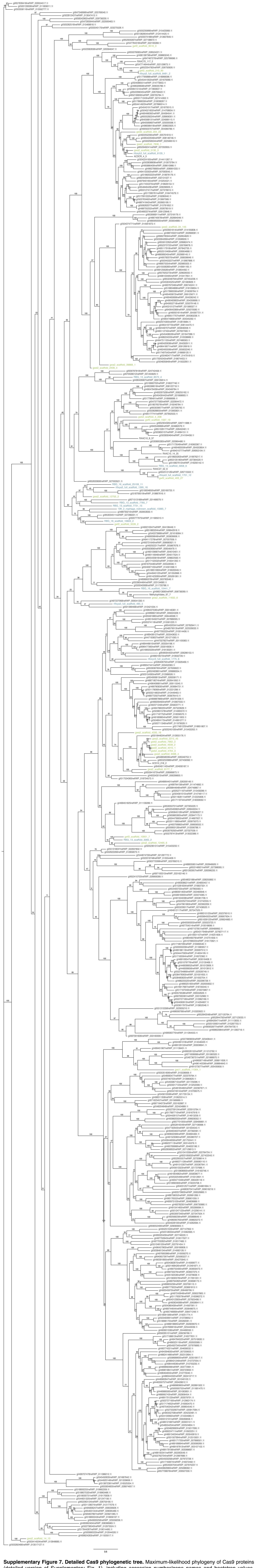

Supplementary Figure 7. Detailed Cas9 phylogenetic tree. Maximum-likelihood phylogeny of Cas9 proteins (detailed version of Supplementary Fig. 1), including accession number/gene names and bootstrap values. Colors represent the source of the Cas9 proteins. Grey: RefSeq, green: Rifle groundwater metagenomic samples, blue: other Rifle metagenomic samples. Alignment used to produce this tree is available in Supplementary Data 7.
